# Preface for the Special Issue on the Exploration of the Multifaceted Roles of Glycosaminoglycans: GAGs

**DOI:** 10.3390/biom11111630

**Published:** 2021-11-04

**Authors:** Dragana Nikitovic, Serge Pérez

**Affiliations:** 1 Laboratory of Histology-Embryology, School of Medicine, University of Crete, 71003 Heraklion, Greece; 2 University Grenoble Alpes, CNRS, CERMAV, 38000 Grenoble, France; serge.perez@cermav.cnrs.fr

Glycosaminoglycans (GAGs) are linear, anionic polysaccharides that consist of repeating disaccharides of hexosamine and hexuronic acid. The exception to this is keratan sulfate, whose building blocks consist of hexosamine and galactose. Differences in the primary disaccharide unit structure regarding uronic acid and hexosamine, the number and position of the sulfate residues, the presence of *N*-acetyl and/or *N*-sulfate groups, and the relative molecular mass are evident. All such differences bestow these biomolecules with impressive complexity and diversity. The fine structure of the disaccharide units defines the types of GAGs. These include chondroitin/dermatan sulfate (CS/DS), heparin/heparan sulfate (Hep/HS), and keratan sulfate (KS), as well as non-sulfated hyaluronan (HA) (Figure 1).

GAGs are ubiquitously localized throughout the extracellular matrix (ECM) and to the cell membranes of cells in all tissues. They are either conjugated to protein cores in the form of proteoglycans, e.g., CS/DS, HS, and KS, or as free GAGs (HA and Hep). Through their interaction with proteins, GAGs can affect the cell-extracellular matrix (ECM) and cell–cell interactions, finely modulating ligand-receptor binding and thus chemokine and cytokine activities as well as growth factor sequestration. Thus, GAGs regulate several biological processes under homeostasis; they also participate in disease progression. Recently, significant advances have been made in the analytic, sequencing, and structural characterization of GAG oligosaccharides as well as in GAG profiling in tissues and cells (GAGomics). Moreover, studies focused on the structure/sequence-function relationships of GAGs have resulted in critical novel insights. Furthermore, advances in the characterization of protein–GAG complexes provide invaluable tools to decipher GAG’s roles in the intricate tissue milieu and answer critical questions regarding GAG participation in the molecular basis of disease and embryonic development.

This Special Issue of *Biomolecules*, entitled “Exploring the multifaceted roles of glycosaminoglycans (GAGs)—new advances and further challenges”, features original research and review articles. These articles cover several timely topics in structural biology and imaging; morphogenesis, cancer, and other disease therapy and drug developments; tissue engineering; and metabolic engineering. This Special Issue also includes an article illustrating how metabolic engineering can be used to create the novel chondroitin-like polysaccharide.

A prerequisite for communicating in any discipline and across disciplines is familiarity with the appropriate terminology. Several nomenclature rules exist in the field of biochemistry. The historical description of GAGs follows IUPAC and IUB nomenclature. New structural depictions such as the structural nomenclature for glycan [1] and their translation into machine-readable formats [2,3] have opened the route for cross-references with popular bioinformatics resources and further connections with other “omics”.

## 1. Structure and Imaging

The structural heterogeneity of GAGs complicates the composition and sequence analysis of GAGs. No less than 200 different monosaccharides have been identified; this has resulted in a very high number of disaccharide segments exhibiting high conformational flexibility. In addition, there have also been intrinsic difficulties in establishing the three-dimensional structures of GAGs. Despite these difficulties and recognizing that the shape of molecules is a fundamental principle in chemistry, physics, and biology, scientists are developing experimental and computational tools to elucidate and understand molecular shapes and molecular motions. Elisabeth Whitmore and collaborators [4] report on the development of efficient atomic resolution models using molecular GAG dynamics. They illustrate the outcome of their application for the case of non-sulfated chondroitin, which may provide insights and arguments for the understanding of disciplines where molecular dynamics play a crucial role.

There is a healthy dialogue between computational and experimental endeavors, a typical example being the structural determination of the oligo of GAGS and polysaccharides and their interactions with proteins. Results have accumulated over time due to X-ray single-crystal diffraction methods, X-ray fiber diffractometry, solution NMR spectroscopy, and scattering data. These data have been curated, annotated, and organized before their structuration into a three-dimensional database containing three-dimensional data on GAGs and GAGs–protein complexes retrieved from the PDB [5]. The database includes protein sequences and the standard nomenclature for GAG composition, sequence, and topology. It provides a family-based classification of GAGs that is cross-referenced with glyco-databases with links to UniProtKB via accession numbers. The 3D visualization of contacts between GAGs and their protein ligands is implemented via the protein–ligand interaction profiler (PLIP). The nature of the structure that GAG polysaccharides can adopt, either solid-state or solution, is also reported. Finally, characterized quaternary structures of the complexes improve our understanding as to if and how GAGs participate in long-range, multivalent binding with potential synergy when several chains are involved in interactions.

Molecular interactions involving GAGs are not restricted to proteins. Many authors consider the large GAG polymeric backbones and their chemical properties to be essential features for the rational design of drug delivery and diagnostic systems. Magnetic resonance imaging is an established diagnostic method for which GAGs, when adequately decorated, offer the benefit of contrast enhancers.

The administration of a paramagnetic contrast agent, such as a metal chelate, such as gadolinium diethylene triamine penta-acetic acid (Gd-DTPA), helps visualize relative GAG distribution in vivo. For example, the negative charge of the contrast agent will distribute itself within articular cartilage in a spatially inverse relationship to the concentration of negatively charged GAG molecules. Alfonso Ponsiglione and collaborators [6] explore the range of advantages that could represent fine control over the combination of GAGs and imaging agents in the formulation of novel multifunctional diagnostic probes.

## 2. Morphogenesis and Development

GAGs, as essential constituents of the human glycome, play pivotal roles in a multitude of biological processes during embryonic development and in the maintenance of homeostasis. Such roles can be observed throughout the structural mold of aggregan and the diversity of its decoration by GAGs. The development of vertebrates from a single cell to the generation of various cell types and organs is carried out throughout a synchronized developmental program consisting of the spatial and temporal coordination of specific signaling molecules, including morphogens and growth factors. The importance of specific arrangements of GAG chains on aggrecan in all of its forms is also a primary morphogenetic functional determinant. It provides aggrecan with unique tissue-context-dependent regulatory properties. The versatility displayed by aggrecan in biodiverse contexts is a function of its GAG side chains [7].

The article by Colin-Pierre et al. [8] describes the evolution of heparan sulfate proteoglycans in hair follicles. The heparan sulfate proteoglycan distribution in hair follicles has traditionally been done by conventional histology, biochemical analysis, and immunohistochemistry. The authors use the absorption region that is relevant to sulfation as a spectral marker. This is performed using infrared spectral imaging (IRSI), which has been used intensively for cell (spectral cytology) and tissue (spectral histology) characterization. Supported by Western blot and immunohistochemistry analysis, infrared spectral imaging specifically shows the qualitative and/or quantitative evolution of the GAGs expression pattern between the anagen, catagen, and telogen phases. Moreover, this demonstrates that IRSI could be utilized for GAG cytology and tissue characterization.

## 3. Therapy: GAGs as Targets and Novel Therapy Agents

GAGs are essential ECMs and cell membrane components and are extensively altered under various pathological conditions, including cancer. Indeed, during disease progression, the fine GAG structure and expression change in a manner that is associated with disease evolution. Furthermore, pathological conditions are characterized by the extent of GAG remodeling that is either due to the increased expression of glycosidases or to a chemical reaction with elevated, radical oxygen species. Specific disease-dependent GAG alterations have been identified as druggable entities, with industry and academic research efforts examining their potential in drug development. Berdiaki et al. [9] discuss the up-to-date developments of implementing GAG disease-dependent changes in two directions: (i) utilizing GAGs as the targets of therapeutic strategies and (ii) employing GAG specificity and excellent physicochemical properties for the targeted delivery of cancer therapeutics.

Faria-Ramos et al. [10] specifically focus on the role of HS in carcinogenesis. Due to HS’s well-established regulation of critical cellular receptors and respective downstream signaling pathways and the aberrant expression of HS in tumor tissue, these GAGs have been characterized as modulators of malignant features. This review article highlights the significant clinical potential of HS to improve both the diagnosis and prognosis of cancer, either as HS-based biomarkers or as therapeutic targets [10].

GAG functions are implicated in inflammatory processes. Notably, cardiovascular disease propagation and the inflammatory status of tissues are closely correlated. The treatment of endothelial cells with the cytokine TNF-α, which is known to be increased in obese patients and has been reported to induce cardiometabolic diseases, strongly affects the expression patterns of hyaluronan and the HS-containing proteoglycans known as syndecans [11]. These changes seem to facilitate the onset of a pathological state by altering (i) the endothelial barrier properties, (ii) increasing HA in the pericellular coat and the possibility of consequent monocyte recruitment from the blood; or (iii) altering the sulfation pattern of membrane-bound HS, which can cause modifications to the endothelium response to growth factors and cytokines. Therefore, the authors confirm the critical role of ECM components such as GAGs in disease progression.

Matrix metalloproteinases (MMPs) are endopeptidases that are able to cleave both matrix and non-matrix proteins. MMPs activity and the resulting extracellular matrix remodeling are increased in acute and chronic diseases and are correlated with disease pathogenesis. Thus, the enhanced activity of MMP-8 facilitates the progression of various pathologies, including atherosclerosis, pulmonary fibrosis, and sepsis. Since natural GAGs are known to modulate the functions of various MMPs, the synthetic non-sugar mimetics of GAGs have been hypothesized to inhibit MMP-8 activity. The strategy of Moria and Desai [12], upon screening a library of 58 synthetic, sulfated mimetics consisting of a dozen scaffolds, led to the identification of sulfated benzofurans and sulfated quinazolinones as promising inhibitors of MMP-8. Interestingly, this work provides the first proof that the sulfated mimetics of GAGs could lead to potent, selective, and catalytic activity-tunable, small molecular inhibitors of MMP-8.

Due to the lack of blood vessels and the consequently limited bioavailability of oxygen and nutrients, articular cartilage has restricted regenerative capacity, resulting in frequent degenerative disease in older individuals. Therefore, therapeutic strategies limiting or halting the progression of cartilage destruction are an unmet health need. Perlecan, a multifunctional HS proteoglycan, promotes embryonic cartilage development and stabilizes mature tissue. Using immunohistochemistry, Garcia et al. [13] showed a pericellular and diffuse matrix staining pattern for perlecan in both natural and cell-therapy-repaired cartilage. This observation was related to whether the morphology of the newly formed tissue was hyaline cartilage or fibrocartilage. In addition, immunostaining was significantly more enhanced in these repair tissues for perlecan than it was for normal age-matched controls and was sensitive to heparanase treatment. Thus, the modulation of HS could be helpful in the treatment of degenerative cartilage disease.

A novel, interesting therapeutic function of heparin has been shown by Lantero et al. [14]. Indeed, these authors report an antimalarial activity of heparin. Innovative antimalarial strategies are urgently needed as plasmodium parasites continue to express increased resistance to the available drugs that have been developed against plasmodium parasites. Heparin delivered in membrane feeding assays together with *Plasmodium berghei*-infected blood of *Anopheles stephensi* mosquitoes was shown to inhibit the parasite’s ookinete–oocyst transition by binding the ookinetes. The inhibition of the parasite life-cycle by heparin might represent a new antimalarial strategy for rapid implementation and is an excellent example of the ubiquitous use of these multifaceted molecules.

## 4. Biomarkers

Because the expression of GAGs and their fine structure are markedly altered in disease, these features could have an essential clinical implementation. Malignant pleural mesothelioma (MPM) is a highly aggressive and therapy-resistant pleural malignancy with poor prognoses and short patient survival. When patients’ pleural infusion was analyzed by a Luminex multiplex assay, syndecan-1 (SDC-1) and MMP-7 levels were significantly lower, whereas mesothelin and galectin-1 levels were significantly higher in malignant mesothelioma effusions compared to in adenocarcinoma. Javadi et al. [15] suggest that MMP-7, shed SDC-1, mesothelin, and galectin-1 can be diagnostic and that VEGF and SDC-1 are prognostic markers in MPM patients. Indeed, this study confirms the vital role of ECM components in malignant disease progression.

## 5. Tissue Engineering and Biomaterial

GAGs are native components of the ECM that drive cell behavior and control the microenvironment surrounding cells, making them promising therapeutic targets for many diseases. Recent studies have shown that the recapitulation of cell interactions with ECM is critical in tissue engineering, which aims to mimic and regenerate endogenous tissues. Because of this, the incorporation of GAGs to drive stem cell fate and to promote cell proliferation in engineered tissues has gained increasing attention. This review article [16] summarizes the role of glycosaminoglycans in tissue engineering and their recent use in these constructs. In addition, the evaluation of the general research trends in this niche offers insight into future research directions in this field.

Hyaluronan displays such properties as biocompatibility, biodegradability, high viscoelasticity, and immunoneutrality, making it attractive for biomedical and pharmaceutical applications. Furthermore, from the standpoint of physical properties, the polyelectrolyte nature of negatively charged hyaluronan provides a way to create new high-performance complexes. One such complex occurs when hyaluronan self-assembles with a positively charged lactose-modified chitosan. The authors of this investigation [17] show that the complex that is formed has a monodisperse molecular weight distribution and a high viscosity and is susceptible to enzymic degradation by hyaluronidase and lysozyme. Due to the wide range of applications in biomedicine and biotechnology, the development of such polyelectrolyte complexes is of scientific and biotechnological interest.

The conjunction of the wide range of biological activities and unique physicochemical properties confer a distinctive place as an implantable biomaterial used in orthopedics and traumatology to hyaluronan. Infections related to implanted medical devices depend on the bacterial capability to establish highly structured multilayered biofilms on artificial surfaces. One way to prevent such peri-implant infection is to apply an implanted biomaterial, defensive antibacterial coating (DAC), which can act as a resorbable barrier that delivers local antibiofilm and antibacterial compounds. The copolymer of hyaluronic acid and poly-d, l-lactic acid produces a hydrogel that retains the hydrophobic character of the poly-d, l-lactide sidechains and the hydrophilic character of a hyaluronic acid backbone. The suitability of such a hydrogel depends on the stability and degradation of both the hyaluronan backbone and the polylactic chains over time and temperature. T. Guzzo and her collaborators [18] performed chromatographic analysis and explored the suitability of diffusion-ordered NMR spectroscopy to characterize the outcome of the biomaterial over time in physiological conditions.

## 6. Metabolic Engineering

Bacterial cells exhibit a wide diversity of capsular polysaccharides that constitute the cell surface of the outer membrane and that mediate interactions with the environment. The capsules are often composed of GAG-like polymers. The structural similarity of microbial capsular polysaccharides to these biomolecules makes these ideal bacteria candidates for non-animal GAG-derived products. It is true that the capsular polysaccharide of *Escherichia coli* K4, the chondroitin synthase polymerase, KfoC, synthesizes a chondroitin-like polysaccharide. While exploring novel methods and conditions to produce chondroitin via metabolic engineering, Leroux and her colleagues expressed KofC in a recombinant strain of *Escherichia coli* deprived of 4-epimerase activity [19]. They realized that KfoC could polymerize a GalNAc-free polysaccharide, giving rise to a novel GAG that they call chondbiuronan.

## 7. Conclusions

While the authors of these articles discussed the multifaceted features of GAGs, they are all well motivated by specific applications in biology and medicine and will develop appropriate tools that are likely to be important in structuring a large amount of available data and opening the field to cross-disciplinary endeavors. We hope that these articles will not only provide timely case studies but that they will also form the basis of a series of questions aimed at answering the broader question of what remains to be solved about GAGs?

## Figures and Tables

**Figure 1:**
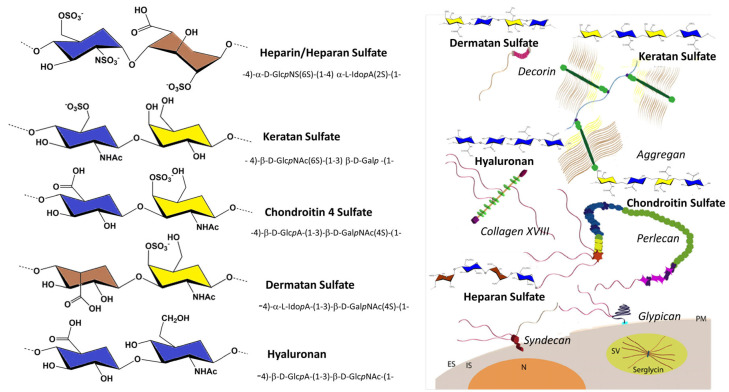
Cartoon representation of the chemical constitution of the five families of GAGs and of six categories of proteoglycans (aggrecan; decorin, perlecan, and collagen; glypican; and syndecan and serglycin). ES, extracellular; IS, intracellular; N, nucleus; SV, secretory vesicle. (Adapted from K. Rodgers, J.D. San Antonio, O. Jacenko. Dev Dyn. 2008, 237, 2622–2642).

## Data Availability

Not applicable.
